# Resources to Support Canadian Nurses to Deliver Virtual Care: Environmental Scan

**DOI:** 10.2196/53254

**Published:** 2024-08-13

**Authors:** Manal Kleib, Antonia Arnaert, Lynn M Nagle, Elizabeth Mirekuwaa Darko, Sobia Idrees, Daniel da Costa, Shamsa Ali

**Affiliations:** 1 Faculty of Nursing University of Alberta Edmonton, AB Canada; 2 Ingram School of Nursing McGill University Montreal, QC Canada; 3 Faculty of Nursing University of New Brunswick Fredericton, NB Canada

**Keywords:** virtual care, digital health, nursing practice, environmental scan, telehealth, nurses, Canada, health care

## Abstract

**Background:**

Regulatory and professional nursing associations have an important role in ensuring that nurses provide safe, competent, and ethical care and are capable of adapting to emerging phenomena that influence society and population health needs. Telehealth and more recently virtual care are 2 digital health modalities that have gained momentum during the COVID-19 pandemic. Telehealth refers to telecommunications and digital communication technologies used to deliver health care, support health care provider and patient education, and facilitate self-care. Virtual care facilitates the delivery of health care services via any remote communication between patients and health care providers and among health care providers, either synchronously or asynchronously, through information and communication technologies. Despite nurses’ adaptability to delivering virtual care, many have also reported challenges.

**Objective:**

This study aims to describe resources about virtual care, digital health, and nursing informatics (ie, practice guidelines and fact sheets) available to Canadian nurses through their regulatory and professional associations.

**Methods:**

An environmental scan was conducted between March and July 2023. The websites of nursing regulatory bodies across 13 Canadian provinces and territories and relevant nursing and a few nonnursing professional associations were searched. Data were extracted from the websites of these organizations to map out educational materials, training opportunities, and guidelines made available for nurses to learn and adapt to the ongoing digitalization of the health care system. Information from each source was summarized and analyzed using an inductive content analysis approach to identify categories and themes. The Virtual Health Competency Framework was applied to support the analysis process.

**Results:**

Seven themes were identified: (1) types of resources available about virtual care, (2) terminologies used in virtual care resources, (3) currency of virtual care resources identified, (4) requirements for providing virtual care between provinces, (5) resources through professional nursing associations and other relevant organizations, (6) regulatory guidance versus competency in virtual care, and (7) resources about digital health and nursing informatics. Results also revealed that practice guidance for delivering telehealth existed before the COVID-19 pandemic, but it was further expanded during the pandemic. Differences were noted across available resources with respect to terms used (eg, telenursing, telehealth, or virtual care), types of documents (eg, guideline vs fact sheet), and the depth of information shared. Only 2 associations provided comprehensive telenursing practice guidelines. Resources relative to digital health and nursing informatics exist, but variations between provinces were also noted.

**Conclusions:**

The use of telehealth and virtual care services is becoming mainstream in Canadian health care. Despite variations across jurisdictions, the existing nursing practice guidance resources for delivering telehealth and virtual care are substantial and can serve as a beginning step for developing a standardized set of practice requirements or competencies to inform nursing practice and the education of future nurses.

## Introduction

### Background

Telehealth refers to the “delivery and facilitation of health and health-related services including medical care, provider and patient education, health information services, and self-care via telecommunications and digital communication technologies. Examples of the technologies used in telehealth include, but are not limited to, live video conferencing, mobile health apps, ‘store and forward’ electronic transmission, and remote patient monitoring.” [1]. During the COVID-19 pandemic, a transition to virtual care became necessary to protect the public and ensure the continuity of health services. Virtual health denotes the facilitation of the delivery of care services through any remote interactions between patients and health care providers and among health care providers themselves, whether synchronous or asynchronous, using information and communication technologies (ICTs) [2]. Various technologies were applied to facilitate virtual care such as SMS text messaging and email; phone; mobile health applications; electronic medical records; chatbots; remote monitoring technologies; and telecommunication applications such as Zoom (Zoom Video Communications), Skype (Skype Communications), FaceTime (Apple Inc), and WhatsApp (WhatsApp LLC) for video consultations [3-9]. Both modalities are subsumed under the umbrella term of digital health, which denotes the proper use of technology to improve the health and wellbeing of people at all levels.

Nurses providing care across a diversity of health care settings had to adapt to virtual care delivery. Despite their engagement and adaptability to this new form of care, many nurses also reported challenges. Those who have not previously used digital health technologies to deliver care had to quickly adapt and learn new skills, and some reported receiving limited guidance or best practice guidelines on how to provide care through these new modalities [10-13]. Before the COVID-19 pandemic, the most recent survey of practicing Canadian nurses’ use of technology identified that only 60% of nurses surveyed about the use of virtual care reported having adequate knowledge and skills to use these technologies [14]. A secondary analysis comparing data from the 2017 and 2020 versions of this survey identified that virtual care was predominantly delivered by nurse practitioners. Several factors were found to predict Canadian nurses’ use of virtual care, including their professional designation, perceived quality of care in the facility where they worked, the type of electronic record used, their perceptions of the quality of care they delivered through virtual care technologies, and their perceptions of the skills and knowledge needed to use these technologies [15]. In addition to gaps among practicing nurses, research has also identified that while new graduate nurses have high digital literacy skills, they struggle to understand the broad spectrum of digital health technologies and their applications in health care [16]. Other studies identified that digital competence, organizational support, prior education about technology, and ongoing training and support are essential factors for effective and safe use and adoption of health information technologies [17-20].

Nurses are integral to the successful implementation and use of technology in health care. Currently, there are >459,000 regulated nurses represented by the Canadian Nurses Association (CNA) at the national level [21]. Provincial regulatory associations also exist across all 13 Canadian provinces and territories. As part of their mandate, nursing regulatory bodies address the registration requirements, standards for education and practice, code of ethics, and continuing competency to ensure nurses are qualified and competent to provide safe, ethical, and evidence-informed practice as well as are prepared to respond to emerging phenomena that influence population health needs and nursing professional practice roles [22,23]. They also develop documents (eg, practice guidelines and practice directives) that outline the activities the regulated nurses are authorized to perform [23]. Professional nursing associations are available to all nurses, nursing students, and nurse retirees, enabling these groups to have a voice in the policy and advocacy work related to profession-wide and societal issues that may have broader impacts on health and health care. In addition to these associations, nursing specialty practice organizations and interest groups serve to provide resources and support for professional development in a specialty practice area such as critical care [24]. Considering the expanded and ongoing use of virtual care and for nurses to thrive when providing care in this increasingly digitalized health care environment, it is important to understand how professional and regulatory nursing associations are contributing to supporting nurses in this area and what knowledge gaps exist in offering necessary support, training, and professional guidance for optimal nursing practice with technology. Addressing this knowledge gap could serve to encourage nursing regulatory and professional bodies to update existing resources or develop new ones, which may consequently contribute to motivating nurses to develop their competency in digital health and virtual care.

### Objective

This study aims to describe resources about virtual care, digital health, and nursing informatics (ie, practice guidelines and fact sheets) available to Canadian nurses through their regulatory and professional associations.

## Methods

### Overview

This study used an environmental scan method [25,26]. An environmental scan is often undertaken when there are emerging issues that necessitate an immediate investigation to determine their potential impact. Environmental scans originated in the business industry as a tool to inform strategic planning and policy decision-making by gathering, interpreting, and using information about the internal and external environment of an organization to guide future actions. Frameworks such as Strengths, Weakness, Opportunities, and Threats analysis or Political, Economic, Social, Technological, Legal, and Environmental are often used to guide this process [25,26]. In health care, environmental scans serve to collect, organize, and analyze information on issues in health care to make evidence-informed decisions, guide program planning, and inform public policy decisions [25]. Environmental scans are also different from other systematic literature searches such as scoping or systematic reviews and may incorporate different and diverse data sources and approaches for collecting information to answer questions of interest. Although the use of environmental scanning has increased in health service research, there is limited methodological guidance on its application. However, because scanning the environment can be extensive, determining the most relevant information sources and outlining the scope of the scan by identifying clear objectives is recommended [25]. In this project, the environmental scan was guided by the following steps: (1) determining the scope of the environmental scan and data sources to be included, (2) scanning resources identified for information that is relevant to the focus of the research, and (3) analyzing and summarizing the information to identify strengths and limitations.

**Figure 1:**
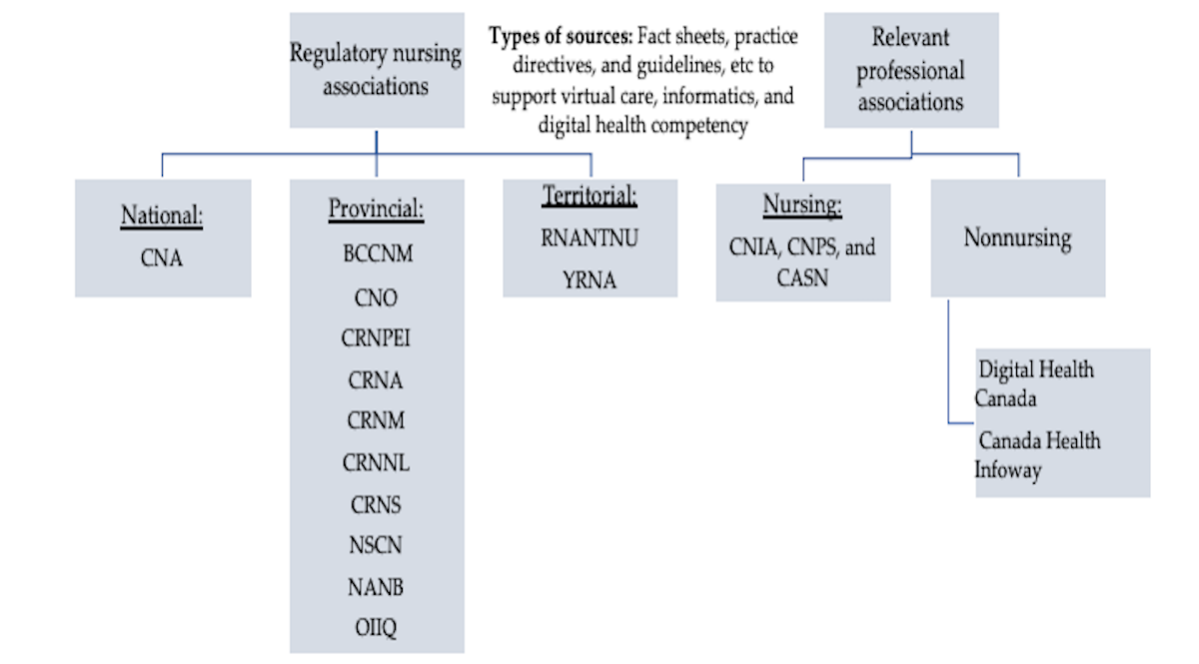
Visual representation of the data sources used in the environmental scan. BCCNM: British Columbia College of Nurses and Midwives; CASN: Canadian Association of Schools of Nursing; CNA: Canadian Nurses Association; CNIA: Canadian Nursing Informatics Association; CNO: College of Nurses of Ontario; CNPS: Canadian Nurses Protective Society; CRNA: College of Registered Nurses of Alberta; CRNM: College of Registered Nurses of Manitoba; CRNNL: College of Registered Nurses of Newfoundland and Labrador; CRNPEI: College of Registered Nurses and Midwives of Prince Edward Island; CRNS: College of Registered Nurses of Saskatchewan; NANB: Nurses Association of New Brunswick; NSCN: Nova Scotia College of Nursing; OIIQ: Ordre des Infirmières et Infirmiers du Québec; RNANTNU: The Registered Nurses Association of the Northwest Territories and Nunavut; YRNA: Yukon Registered Nurses Association.

The environmental scan was conducted between March 2023 and July 2023 to identify and retrieve pertinent information and resources available to guide nursing practice or education about virtual care, digital health, and informatics for Canadian nurses. A search of the websites of national, provincial, and territorial Canadian nursing regulatory bodies and the websites of selected professional nursing associations, nursing specialty practice organizations, and some nonnursing organizations and associations was conducted. To locate the relevant available data on the websites of each organization and association, we used different search terms such as *digital health, nursing informatics (NI), virtual care, telehealth, telenursing, telemedicine*, and *nurses*. The authors then examined what was reported in each record identified on each association website by reviewing the content, date, relevance, and the type of the resource. Nursing unions were excluded from this scan because their mandate is not related to professional development or regulation. Only information published in English was reviewed. Figure 1 summarizes the sources of data used in this scan.

### Data Analysis

We applied an inductive qualitative content analysis approach to categorize data extracted from the retrieved documents [27,28]. Inductive content analysis is an appropriate approach for descriptive qualitative analysis. It focuses on identifying easily observable items or data without the need to discern intent or identify deeper meaning. It uses a systematic approach to transform large amounts of data into a highly organized and concise summary of results that can be grouped into codes, categories, and themes. The Provincial Health Service Authority (PHSA) Virtual Health Competency Framework was also used to support the analysis and interpretation of the data [29]. Two reviewers independently completed the process of data abstraction and coding, and data were compared for accuracy to ensure consistency and rigor in the abstraction process.

### Ethical Considerations

As per the ethics requirements at the University of Alberta [30], we did not seek ethics clearance because the research did not involve collecting input or information from human participants. We primarily relied on publicly available information that is legally accessible to the public.

## Results

### Overview

A total of 17 websites were included in this scan, of which 13 (76%) were for regulatory bodies (n=1, 8% national; n=10, 77% provincial; and n=2, 15% territorial), 2 (12%) were professional associations, and 2 (12%) were part of the relevant nonnursing organizations. On the basis of the nature of the information provided in the retrieved documents or resources, seven overarching themes were identified: (1) types of resources available about virtual care, (2) terminologies used in virtual care resources, (3) currency of virtual care resources identified, (4) requirements for providing virtual care between provinces, (5) resources through professional nursing associations and other relevant organizations, (6) regulatory guidance versus competency in virtual care, and (7) resources about digital health and NI. These are discussed in detail in the subsequent sections.

### Types of Resources Available About Virtual Care

Different types of guidance documents such as fact sheets, practice directives, guidance, practice guidelines, and general guidelines were identified on the web pages of nursing regulatory bodies (Table 1). The CNA issued a telehealth fact sheet in 2017 and did not provide national level guidance pertaining to telenursing practice or virtual care; however, it has identified virtual care as a priority advocacy area. Some associations provided documents in the form of fact sheets, guidance, and practice directives (College of Nurses of Ontario [CNO], College of Registered Nurses of Manitoba [CRNM], and College of Registered Nurses and Midwives of Prince Edward Island [CRNPEI]). Only 2 associations published telenursing practice guidelines for registered nurses and nurse practitioners (Nurses Association of New Brunswick [NANB] and the Nova Scotia College of Nursing [NSCN]). Some associations supplemented their guidelines with educational resources such as practice scenarios, case studies, and YouTube videos (NSCN and British Columbia College of Nurses and Midwives [BCCNM]), and some associations provided resources to support nurses’ mental health and well-being (CNO). Some regulatory bodies provided external links to organizations, such as the Canadian Nurses Protective Society (CNPS), on their website.

**Table 1:** Websites of nursing and related organizations offering virtual care resources.

Organization			Virtual care		Concepts emphasized and general assessment
**National and provincial regulatory nursing associations**					
	CNA^a^	Virtual care as an advocacy priority (2023) Telehealth fact sheet (2017)		Virtual care has been identified as an advocacy priority Telepractice fact sheet has not been updated since 2017 No further guidance provided during or after the pandemic	
	BCCNM^b^	Virtual care learning resource		A unique web page providing a definition, FAQs^c^, case study about providing virtual care to clients outside British Columbia, links to related standards, policies, guidelines, professional standards, and additional resources on virtual health (a handbook and toolkit)	
	CNO^d^	Telepractice fact sheet (2023) COVID-19 practice resources		The document provides brief information on the expectations of nurses providing care both within and outside Ontario. In addition, it is stated that the telepractice guideline is under review. Furthermore, there are links to resources, such as the scope of practice, code of conduct, therapeutic nurse-client relationship, documentation, and confidentiality and privacy: personal health information Specific resources for COVID-19, such as self-care fact sheets and other information and tips to support mental health for nurses	
	CRNPEI^e^	Practice directive—telehealth nursing practice (2019) Practice directive—technology in practice (2020)		Telehealth practice directives: address regulatory requirements Technology in nursing practice: provide guidance on aspects of virtual care Concepts addressed: therapeutic nurse-patient relationship, competencies beyond basic nursing program, consent, privacy, confidentiality, and ethical and legal considerations There are links to external resources (eg, CNPS^f^ infoLAW page that has information on relevant topics such as confidentiality of health information and social media)	
	CRNA^g^	Telepractice and virtual care (2020)		Regulatory guidance to RNs^h^ and NPs^i^ on licensing requirements, indicating that they must be licensed in the jurisdiction they are providing care, regardless of the practice setting RN entry-level competencies include indicators about nursing informatics	
	CRNM^j^	Practice expectations spotlight: telepractice or virtual care (2020) Guidance on telepractice (2020) Telepractice (2021)		A news item providing generic guidance regarding risks associated, focusing on miscommunication, privacy breaches, and poor coordination of care Additional resources on the page link to CNPS Telephone Advice (2009), but no link or listing of more current CNPS resources about virtual care A guide providing information on general performance expectations, informed consent, privacy, safety considerations and adverse events, safety expectations, competency, and documentation and billing for health care providers including nurses An information sheet of telepractice on professional practice both within and outside of Manitoba	
	CRNNL^k^	Virtual nursing practice (2020) Fact sheet: virtual nursing licensure requirements (2022)		A document articulating the practice expectations required of RNs and NPs participating in virtual nursing practice and examples of virtual care technologies A fact sheet that addresses licensure requirements for engaging in virtual nursing practice.	
	CRNS^l^	Tag: telehealth—nursing use of ICTs^m^ (2020)		General and regulatory guidance on telehealth and nursing use of ICT technologies. Concepts briefly addressed the following: upholding standards, competencies, code of ethics, clinical knowledge, clinical judgment, communication, documentation skills, and nurse-client relationship	
	NSCN^n^	Telenursing practice guidelines for nurses (2019, last revised in February 2023) Practice scenarios and FAQs for nurses working with virtual MDs^o^ or NPs (2023) YouTube video (12 minutes) Telenursing online education module Fact sheet: LPN^p^ insurance program—providing telehealth Glossary (2023) COVID-19 information and resources including social media use		NSCN has the most recent and updated detailed guidelines on telenursing practice for RNs and NPs. It has also recently published a glossary of associated terms related to virtual care Core concepts addressed the following: telenursing; professional practice; competencies; risk management; informed consent; confidentiality; therapeutic nurse-client relationship; communication; documentation; virtual care; and telehealth services including videoconferencing, phone call, web-based patient portals, email and SMS text messaging, and other electronic communications Following the outbreak of the pandemic, it provided detailed information about social media use	
	NANB^q^	Telenursing practice guidelines (February 2023) FAQ: what are the registration requirements to provide telenursing care in NB?		The toolkit provides guidance and resources to assist nurses and employers in providing safe, competent, and ethical telenursing care Core concepts addressed the following: telenursing practice, accountability, competency, client-centered care, legal requirements, confidentiality, client safety, custodian of record, competency, evidence-informed practice, communication, documentation, nurse-client relationship, informed consent, and risk management Additional guidance on regulatory requirements is explored in the FAQ component	
	OIIQ^r^	—^s^		Information on this website is in French and therefore was not verified	
	RNANTNU^t^	FAQs: telehealth News page provides hyperlinks to external resources		FAQs provide general guidance on licensure and providing care in different jurisdictions. News page provides hyperlinks to information through other organizations, such as CASN^u^ and CNPS, that provide information about virtual care	
	YRNA^v^	None		No virtual care guidelines. Documentation guidelines have not been updated since 2013	
**Relevant professional nursing associations**					
	CNIA^w^	None		The 2023 conference (Accelerating Digital Care Capacity in Nursing) focused on how virtual care influenced nursing practice	
	CNPS	InfoLAW: Telepractice (2020) FAQ providing information about 12 things to consider before joining a virtual care practice (2020, updated in 2022)		The telepractice InfoLAW guide provides a general overview of telepractice addressing aspects related to privacy, risk management, jurisdictional considerations with links to regulatory bodies’ telepractice resources, and a case study supplemented with a quiz. It does not replace regulatory requirements. Some resources are accessible publicly, but membership is needed to access resources such as case studies and quizzes, which require registration with an access code.	
**Relevant nonnursing organizations**					
	CHI^x^	Clinician change virtual care toolkit (2022)		CHI has substantive resources about digital health—refer to the main web page. A detailed guide is available for new and experienced users or clinicians on how to provide safe and high-quality virtual care. The toolkit provides guidance to new and experienced users on how to provide safe and high-quality virtual care.	
	Digital Health Canada	Virtual care in Canada: Lexicon (2021) Virtual care in Canada: maturity model framework (2021)		This organization has dedicated and extensive resources and certification for health informatics professionals.	

^a^CNA: Canadian Nurses Association.

^b^BCCNM: British Columbia College of Nurses and Midwives.

^c^FAQ: frequently asked question.

^d^CNO: College of Nurses of Ontario.

^e^CRNPEI: College of Registered Nurses and Midwives of Prince Edward Islan.

^f^CNPS: Canadian Nurses Protective Society.

^g^CRNA: College of Registered Nurses of Alberta.

^h^RN: registered nurse.

^i^NP: nurse practitioner.

^j^CRNM: College of Registered Nurses of Manitoba.

^k^CRNNL: College of Registered Nurses of Newfoundland and Labrador.

^l^CRNS: College of Registered Nurses of Saskatchewan.

^m^ICT: information and communication technology.

^n^NSCN: Nova Scotia College of Nursing.

^o^MD: medical doctor.

^p^LPN: licensed practical nurse.

^q^NANB: Nurses Association of New Brunswick.

^r^OIIQ: Ordre des Infirmières et Infirmiers du Québec.

^s^Not available.

^t^RNANTNU: The Registered Nurses Association of the Northwest Territories and Nunavut.

^u^CASN: Canadian Association of Schools of Nursing.

^v^YRNA: Yukon Registered Nurses Association.

^w^CNIA: Canadian Nursing Informatics Association.

^x^CHI: Canada Health Infoway.

### Terminologies Used in Virtual Care Resources

Across the available resources, regulatory bodies used different terms and to some extent interchangeably, including telehealth, telepractice, telepractice nursing, virtual care, and technology. In their fact sheet, the CNA used the term telehealth; similarly, the Registered Nurses Association of the Northwest Territories and Nunavut (RNANTNU) and the College of Registered Nurses of Saskatchewan (CRNS) used telehealth on their web pages to describe the care provided using ICTs. The CRNPEI, in their practice directive document, used the terms telehealth nursing practice and technology in practice, whereas the CNO, in their fact sheet, used the term telepractice. In the CRNM guidance on telepractice, the term telepractice was used, but both the terms telepractice and virtual care were used on the web page. In describing telepractice, the CRNM also indicated that telepractice is also known as virtual care. Furthermore, the College of Registered Nurses of Newfoundland and Labrador (CRNNL), in a document provided on their website, used the term virtual nursing practice. The practice guidelines and guidelines provided by the NSCN and NANB, respectively, used the term telenursing and telenursing practice. At the same time, the College of Registered Nurses of Alberta (CRNA), on their web page, used the terms telepractice and virtual care simultaneously.

Some associations, including CNO, BCCNM, NANB, and CRNPEI, provided links to nurses’ scope of practice and practice standards of practice documents on their web pages and in the documents. This implies that when providing virtual care or telehealth, nurses are also expected to abide by these overarching practice requirements; however, these documents do not explicitly discuss or guide nurses in understanding virtual care. Associations such as CNO, NANB, and NSCN had links to other registered nurse professional practice resources (eg, scope of practice, privacy of health information, code of ethics, code of conduct, and documentation practices) that are included in these documents, but these also did not have specific guidance on telehealth or virtual care.

### Currency of Virtual Care Resources Identified

The information provided in the resources screened was evaluated using the Currency, Relevance, Authority, Accuracy and Purpose checklist [31]. This method evaluates different dimensions to ensure the credibility of a source of information. This scan used the description under the Currency, which examines the date of publication and the last date updated as well as the relevance of the topic or information, that is, determining if the information addresses current events. Relating to the description provided by the Currency, this scan focused on the COVID-19 pandemic, virtual care and nursing regulatory bodies, and professional associations’ websites, as these sources are more likely to provide up-to-date and accurate information to their membership.

In 2023, the NSCN and NANB provided practice guidelines and guidelines to direct the provision of care in a safe, competent, and ethical manner. The current nature of these documents suggests that the NSCN and NANB noticed the increased use of virtual care and the importance of providing detailed guidelines to assist nurses in increasing their knowledge and capacity in telenursing. In addition, the CNO provided an updated version of their fact sheet on telepractice in 2023 while the guideline was being reviewed. Some of the associations have not updated their documents and information in recent times. The CNA last updated their telehealth fact sheet in 2017; CRNPEI updated their practice directive for telehealth nursing practice in 2019 and technology in practice in 2020; CRNA had information on telepractice and virtual care information on their web page dated 2020; CRNM guidance on telepractice was published in 2020, an informational document on telepractice was also published in 2021, and the practice expectations spotlight—telepractice or virtual care—was posted in 2020; CRNNL published virtual nursing practice guidelines in 2020; and CRNS made the tag telehealth—nursing use of information and communication technologies available in 2020. As noted in the publication date of these documents, the surge for developing guidance about telehealth and virtual care occurred mainly during the COVID-19 pandemic.

### Requirements for Providing Virtual Care Between Provinces

Regulatory bodies in 12 provinces provided a statement on the legislative requirement of practicing telehealth or virtual care in and out of the nurse’s jurisdiction. Information from the Quebec’s nursing regulatory body, which had the information in French, was not verified. The fundamental requirement, according to the regulatory bodies, is that nurses must have a valid registration in their jurisdiction. Most of the regulatory bodies, BCCNM, CNO, CRNPEI, CRNA, CRNM, CRNNL, NSCN, and NANB, described the legislative requirements around providing telehealth or virtual care when care is provided in the nurses’ jurisdictions and across jurisdictions. The CRNNL, CRNM, CRNA, and CNO also provided additional information on the expectations for an out-of-province nurse in providing telepractice to residents outside their jurisdiction. Because of the variation in the registration requirement and the legal aspects involved, especially for out-of-province nurses, it is important for nurses to contact the appropriate authorities or organizations, such as the CNPS and regulatory bodies, before starting to practice in a virtual environment.

### Resources Through Professional Nursing Associations and Other Relevant Organizations

The CNPS is a not-for-profit society that offers legal advice, risk management services, legal assistance, and professional liability protection to >140,000 nurses registered with it [32]. During the course of the pandemic, the CNPS took the initiative to look up regulatory and legal guidance available about virtual and telehealth practice for nurses in Canada and made these resources more accessible by providing the URL links to information already posted on the provincial nursing regulatory associations’ websites. They also developed some educational resources in the form of case studies for nurses and nursing students to understand different scenarios and possible legal risks associated with virtual nursing practice. Most of these resources were freely accessible in the first 2 years of the pandemic, but now the learning portion of these resources is accessible to CNPS members only. The Canadian Nursing Informatics Association (CNIA) annual conference in 2023 focused on highlighting nurses’ innovations and successes in delivering virtual care and directions toward accelerating nursing digital capacity. Digital Health Canada and Canada Health Infoway published up-to-date key resources related to virtual care and both organizations continue to hold regular webinars to facilitate and guide virtual care delivery.

### Regulatory Guidance Versus Competency in Virtual Care

To ascertain whether current resources could be leveraged to further enhance nurses’ competency in delivering virtual care, we compared the information provided by the regulatory nursing associations in the form of guidelines, practice directives, and fact sheets on telehealth or virtual care with the PHSA Virtual Health Competency Framework for health care providers delivering virtual health, which was developed based on a joint collaboration between the Office of Virtual Health in British Columbia, clinical partners, patients, and family partners. It also applied a comprehensive search of existing literature to identify the domains that reflect the foundational and functional competencies needed to deliver safe care in a virtual environment [29]. As shown in Table 2, only the NANB and NSCN provided comprehensive guidelines, which to a large extent were congruent with most competency indicators proposed in the PHSA framework, although both associations used the term telenursing in their guidelines, as opposed to virtual health. The CRNPEI, in their practice directive, provided some competency expectations for nurses under the section virtual care modalities; however, the competencies identified were related to general technology use and policies without explicitly mentioning virtual care or telehealth. Similarly, the CRNM, in their guidance on telepractice, identified competency in relation to the use of technology and telepractice.

**Table 2:** Comparison between Provincial Health Service Authority (PHSA) virtual health competencies and the existing regulatory nursing guidance.

PHSA virtual health competencies		Nursing associations		
Domains and competencies		NANB^a^	NSCN^b^	CRNM^c^
**Domain 1: virtual health practice requirements—encompasses awareness and understanding of legal, regulatory, and organizational virtual health policies**				
	Demonstrates an awareness of the legal and regulatory requirements and practice standards that inform and guide delivery of virtual health	✓	✓	
	Applies relevant organizational policies and decision support tools for safe and effective virtual health	✓	✓	
**Domain 2: technology for virtual health—encompasses an understanding of how to use organizational virtual health technologies appropriately and safely**				
	Demonstrates the knowledge and skills needed to use virtual health tools	✓	✓	✓
	Demonstrates an awareness and understanding of the privacy, security, and safety features of virtual health tools	✓	✓	
**Domain 3: equity-oriented care for virtual health—encompasses the ability to use equity-oriented care in virtual health practice**				
	Applies principles of equity-oriented care to determine if virtual health can improve access or exacerbate barriers to care	✓		
	Advocates for and leverages resources to ensure access to virtual health	✓		
**Domain 4: delivery of virtual health—encompasses knowledge and capacity to deliver safe, high-quality virtual health**				
	Incorporate virtual health safely and appropriately into clinical practice	✓	✓	
	Apply trauma awareness, cultural humility and sensitivity, and harm reduction in virtual health practice	✓		
	Support patient’s and family’s informed decision-making on the risks and benefits of virtual health	✓	✓	
	Determine what technological supports patients and families need when using virtual health tools	✓	✓	
	Communicate clearly and respectfully in the virtual health environment	✓	✓	
	Demonstrate the skills and judgment needed to safely and effectively complete a virtual health clinical interaction	✓	✓	
	Recognize and respond appropriately to the patients’ emotional, psychological, social, and physical needs in the virtual health environment	✓	✓	
	Provide effective patient and family support and share education and follow-up recommendations in the virtual health environment	✓	✓	
	Integrate into virtual health practice the appropriate referral process and documentation standards to ensure quality care	✓	✓	

^a^NANB: Nurses Association of New Brunswick.

^b^NSCN: Nova Scotia College of Nursing.

^c^CRNM: College of Registered Nurses of Manitoba.

### Resources About Digital Health and NI

Within the Canadian context, NI competency involves the use of digital health tools to support information synthesis in accordance with professional and regulatory standards in care delivery [16]. As shown in Table 3, various resources are available on regulatory websites to strengthen nursing capacity in digital health and informatics. The CNA and CNIA in 2017 developed a joint position statement on NI, emphasizing the importance of embracing NI competencies to advance nursing practice and knowledge development. At the time of this writing, the development of a revised CNA and CNIA position statement is underway. Across the 13 provinces and territories, only 4 provinces had an NI group presence (Ontario, Alberta, Saskatchewan, and Nova Scotia). Some information could not be verified. For example, the New Brunswick NI group has a Facebook account, but the last event posted on this page was in 2017. In addition, there is a web link on the Facebook page with a link to the NI group website, which is inactive. At the time of this writing, the Atlantic Canada Nursing Informatics Chapter has been created as part of CNIA in 2023, including the provinces of New Brunswick, Nova Scotia, Newfoundland and Labrador, and Prince Edward Island. The other association is the Association of Registered Nurses of Manitoba, which includes the Manitoba Nursing Informatics Association as a specialty group; however, the link to the Manitoba Nursing Informatics Association is inactive.

Most regulatory nursing associations included information related to the privacy of personal health information and electronic or paper-based documentation as part of standard practice documents such as the entry-to-practice competencies; however, there is limited discussion of NI or digital health concepts. Several associations, including the CRNS, CRNA, and NANB, provided guidelines for social media and e-professionalism for nurses in 2021 and 2022, including the ethical and professional obligations of nurses. Although the Registered Nurses Association of the Northwest Territories and Nunavut also had a document on social media, this was before the pandemic, and it has not been updated recently. Professional nursing associations, including the CNIA and the Canadian Association of Schools of Nursing, have publicly available educational resources about digital health and NI standards such as “the Nursing Informatics Entry-to-Practice Competencies for Registered Nurses” to guide nursing practice and education. At the time of this writing, the development of a revised NI competencies is underway. Nonnursing organizations, such as including Digital Health Canada and Canada Health Infoway, offer extensive resources related to digital health in Canadian health care as well as health informatics competencies for health care providers. However, currently, it is not known how many nurses are actually using these resources.

**Table 3:** Websites of nursing and related organizations offering digital health and nursing informatics (NI) resources.

Organization			Informatics and digital health		Comments
**National and provincial regulatory nursing associations**					
	CNA^a^	Position statement: NI (2017) Infographic: advancing an essential clinical data set in Canada (2019-present) Fact sheet: privacy of personal health information (2011)		A joint position statement by CNA and CNIA^b^ on the development of NI and competencies A fact sheet on the privacy of personal health information, providing information on federal, provincial, and territorial privacy laws. In addition, information on resources that support the protection of the privacy of personal health information, including the Pan-Canadian Health Information Privacy and Confidentiality Framework, PIPEDA^c^ Awareness Raising Tools Initiative, and CNA’s Code of Ethics for Registered Nurses, is provided.	
	BCCNM^d^	Not available		—^e^	
	CNO^f^	Ontario Nursing Informatics Group		A web page containing information on the activities of the Ontario Nursing Informatics Group in the development and promotion of NI among nurses in Ontario.	
	CRNPEI^g^	Documentation standards (2021)		A practice directive document on documentation standards that describes the accountability and expectations of nurses concerning documentation in all practice settings, irrespective of the documentation method or storage. The major concepts addressed in the document are principles of documentation, confidentiality, importance of documentation, purpose, who has a role in documentation, cosigning and countersigning entries, key elements of nursing documentation, and timing of documentation. In addition, resources on legislation at the federal and provincial levels that affect nursing documentation are provided.	
	CRNA^h^	Social media and e-professionalism: guidelines for nurses (2021) Privacy and management of health information standards (2022) Entry-level competencies for the practice of registered nurses (2019) Documentation standards (2022) Nursing Informatics Association of Alberta		A document providing guidelines on professional and ethical obligations with online presence/social media and competencies expected of entry-level and experienced nurses The responsibilities of managing health information and the requirements of the Health Information Act for custodians and affiliates are also addressed The core concepts addressed in the documentation process are communication, accountability, legal implications, and expected standards for documentation A blogspot providing information on activities of the Nursing Informatics Association	
	CRNM^i^	Manitoba Nursing Informatics Association (link is inactive)		—	
	CRNNL^j^	None		—	
	CRNS^k^	Social media guidelines for RNs^l^ (2021) Documentation guidelines for RNs (2021) General overview of NI to nurse managers of RNs SNIA^m^		A dedicated website on Saskatchewan Nursing Informatics Association with links to associated nursing informatics journals, digital health resources, relevant external resources, professional practice, and CNIA The SNIA provides additional resources and links (publications and links to CNPS and CNO telepractice guidelines), but these do not include their own guidance on virtual care General guidelines for NI practice on social media and documentation and a specific description of NI to nurse managers supervising RNs	
	NSCN^n^	Nova Scotia Nursing Informatics Group		NSCN has established an informatics group that holds monthly meetings and guides nurses on the use of ICT^o^ in health care. The web page provides links to other international, national, and provincial organizations that align with digital health and informatics.	
	NANB^p^	Fact sheet: misinformation and disinformation (April 2023) FAQ^q^: nursing documentation Practice guidelines: social media (2022) New Brunswick Nursing Informatics Group		A practice guide providing information on the overview of social media and the general expected responsibilities of nurses when using the platform Additional information is provided on general nursing documentation with additional guidance on considerations on electronic documentation, how to document telepractice, or nursing care provided virtually The New Brunswick NI group Facebook page has little detail about the group and has no current activity or presence. A new Atlantic chapter (the Atlantic Canada Nursing Informatics Chapter) has recently been created.	
	OIIQ^r^	—		The information on the website is written in French.	
	RNANTNU^s^	Documentation guidelines (2015) Position statement—social media (2015)		Detailed information on general documentation guidelines, which have not been updated since 2015. Concepts addressed are standards related to responsibility and accountability, knowledge-based practice, client-centered service, and public trust Has a document on regulatory expectations for RNs and NPs^t^ and the benefits and risks of using social media	
	YRNA^u^	Documentation guidelines (2013)		Generic information about the documentation process and details with aspects of electronic documentation	
**Professional nursing associations**					
	CNIA	2023 conference—Accelerating Digital Care Capacity in Nursing General resources		One of the conference themes focused on how virtual care influenced nursing practice The resource page provides various informational resources on digital health, informatics within education and practice, NI teaching toolkits, CASN^v^ NI entry-to-practice competencies for RNs, and CNA e-Nursing strategy document	
	CASN	NI entry-to-practice competencies for RNs Dedicated web page for digital health and informatics education		A range of resources, including webinars, toolkits, whiteboard animation videos, research, and web-based self-paced 5 modules. These are primarily targeted at nurse educators and nursing programs. NI competencies update is currently underway.	

^a^CNA: Canadian Nurses Association.

^b^CNIA: Canadian Nursing Informatics Association.

^c^PIPEDA: Personal Information and Electronic Documents Act.

^d^BCCNM: British Columbia College of Nurses and Midwives.

^e^Not available.

^f^CNO: College of Nurses of Ontario.

^g^CRNPEI: College of Registered Nurses and Midwives of Prince Edward Islan.

^h^CRNA: College of Registered Nurses of Alberta.

^i^CRNM: College of Registered Nurses of Manitoba.

^j^CRNNL: College of Registered Nurses of Newfoundland and Labrador.

^k^CRNS: College of Registered Nurses of Saskatchewan.

^l^RN: registered nurse.

^m^SNIA: Saskatchewan Nursing Informatics Association.

^n^NSCN: Nova Scotia College of Nursing.

^o^ICT: information and communication technology.

^p^NANB: Nurses Association of New Brunswick.

^q^FAQ: frequently asked question.

^r^OIIQ: Ordre des Infirmières et Infirmiers du Québec.

^s^RNANTNU: The Registered Nurses Association of the Northwest Territories and Nunavut.

^t^NP: nurse practitioner.

^u^YRNA: Yukon Registered Nurses Association.

^v^CASN: Canadian Association of Schools of Nursing.

## Discussion

### Principal Findings

This environmental scan revealed several strengths and opportunities for improvement. The existing resources available to nurses for providing virtual care are mostly current and have evolved during the course of the COVID-19 pandemic. Despite variations in the types, depth of the information shared, and terms used to describe virtual care and guide nursing practice as described in the existing resources, the availability of such resources indicates that regulatory nursing associations were responsive to the needs of their members and to the evolving phenomena affecting health care and nursing practice in Canada.

Considering the ongoing use of virtual care and the potential risk of reverting to a large-scale use of this form of care delivery should events similar to the COVID-19 pandemic occur in the future, there might be benefits in adopting consistent terminologies as well as practice requirements and competencies. By having formalized standards of practice and competencies related to telehealth, telenursing, or virtual care and constantly updating them to reflect current needs across provinces, this will likely reduce uncertainties among nurses and minimize the tendency for taking a reactive approach to virtual care delivery. Such an approach will also better enable nurses to comprehensively develop their knowledge and competency in providing virtual care, as opposed to seeking information on a need-to-know basis. The PHSA Virtual Health Competency Framework and its compatibility with the existing comprehensive telenursing practice guidelines developed by the NANB and NSCN and the currency of these resources is promising. This framework can be used by regulatory nursing association to complement telenursing practice guidelines as well as by schools of nursing to teach nursing students about virtual health.

Having consensus on the scope of telenursing practice and virtual care across jurisdictions not only enables nurses to better respond to potential future events similar to the scale of the COVID-19 pandemic but also enables them to successfully adapt to the ongoing digital transformation taking place in Canadian health care [33-36]. This is important because upon reviewing available resources aimed at guiding and developing NI competencies as well as supporting nursing practice in digital health, it was found that these resources also varied. For example, some associations provided documentation guidelines to their members, but these were not consistently updated. In addition, they did not specifically address documentation in electronic health records or link to NI competencies.

Nurses represent the largest group of Canadian care providers, and regardless of their professional designation, they are at crossroads with digitalization [33,36]. As such, engaging the nursing workforce and supporting the development of new skills is vitally important to realize the full potential of digital innovations [35]. This will enable nurses to leverage digital technologies to further enhance nursing practice, facilitate accessibility to care, and improve overall health outcomes [33,36]. As part of their professional responsibility and accountability, nurses must also pursue professional development opportunities in digital health and NI offered through their professional associations or other sources as well as to keep abreast with issues and phenomena that influence nursing practice and health care. It is also important that health systems critically reflect on lessons learned from the pandemic, including the benefits and challenges associated with virtual care delivery and proactively planning for what should be done differently going forward. Furthermore, it is incumbent upon health systems investing in digital health technologies to support nurses in adapting to this changing context of health care and to work collaboratively with regulatory and professional nursing associations and schools of nursing to develop digital care capacity among practicing and future nurses. An evidence of the commitment of health care governing authorities at the federal, provincial, and territorial levels to support efforts to increase technology adoption and virtual care is the report released by the Canadian Institute of Health Information, in which they developed a series of case studies based on semistructured interviews with representatives from provinces and territories [37]. These case studies addressed various aspects, including strategy, governance and direction setting, programs, and initiatives, providing an excellent resource to facilitate learning and policy direction regarding virtual care [37]. Similarly, Canadian Association of Schools of Nursing has recently secured funding from Health Canada to sponsor the project “Essential COVID-19 Skills for Graduating and New Nurses” [38]. Throughout the COVID-19 pandemic and continuing to the present, the CNA has engaged in focused advocacy work. This includes recommendations for implementing a pan-Canadian digital health strategy; investing in virtual care to support populations considered vulnerable; ensuring that health workers receive appropriate training; and providing financial support to help jurisdictions deploy essential infrastructure and access reliable, high-speed internet services [39].

### Conclusions

Despite challenges during the COVID-19 pandemic, it also served as a catalyst for expanding access to care through digital health modalities such as telehealth and virtual care. Nursing associations play an important role in regulating and ensuring that nurses provide safe, competent, and ethical care. Considering that nursing practice with telehealth and virtual care has become mainstream, there are opportunities to build on these successes and mitigate potential risks in the future. Work completed to date to inform Canadian nursing practice in a virtual context is substantial and can serve as a beginning step for developing a standardized set of requirements to inform nursing practice in telehealth and virtual care and in the education of future nurses. This environmental scan followed a systematic search and provided important insights into the current state of resources available to nurses in Canada regarding virtual care through their professional and regulatory associations. To our knowledge, no prior work has been done on this topic. This scan may not be applicable to nurses outside Canada.

## Abbreviations

BCCNM: British Columbia College of Nurses and Midwives
CNA: Canadian Nurses Association
CNIA: Canadian Nursing Informatics Association
CNO: College of Nurses of Ontario
CNPS: Canadian Nurses Protective Society
CRNA: College of Registered Nurses of Alberta
CRNM: College of Registered Nurses of Manitoba
CRNNL: College of Registered Nurses of Newfoundland and Labrador
CRNS: College of Registered Nurses of Saskatchewan
NANB: Nurses Association of New Brunswick
NI: nursing informatics
NSCN: Nova Scotia College of Nursing
PHSA: Provincial Health Service Authority
